# Effects of curcumin on the pharmacokinetics of amlodipine in rats and its potential mechanism

**DOI:** 10.1080/13880209.2020.1764060

**Published:** 2020-05-20

**Authors:** Na Jiang, Meicheng Zhang, Xiangzhi Meng, Bin Sun

**Affiliations:** a Department of Emergency, Yidu Central Hospital of Weifang, Weifang, Shandong, China; b Department of Cardiovascular Medicine, Yidu Central Hospital of Weifang, Weifang, Shandong, China

**Keywords:** Drug–drug interaction, CYP3A4, metabolic stability

## Abstract

**Context:**

Hyperlipidaemia and hypertension are often treated together with curcumin and amlodipine. It is necessary to investigate the drug-drug interaction between curcumin and amlodipine.

**Objective:**

The interaction between curcumin and amlodipine was investigated in rats and with rat liver microsomes.

**Methods:**

The pharmacokinetics of amlodipine (1 mg/kg) was investigated in rats with or without curcumin pre-treatment (2 mg/kg), six rats in each group. The metabolic stability of amlodipine was investigated with rat liver microsomes.

**Results:**

Curcumin significantly increased the *C*_max_ (26.19 ± 2.21 versus 17.80 ± 1.56 μg/L), *AUC_(0-t)_* (507.27 ± 60.23 versus 238.68 ± 45.59 μg·h/L), and *t_1/2_* (14.69 ± 1.64 versus 11.43 ± 1.20 h) of amlodipine (*p* < 0.05). The metabolic stability of amlodipine was significantly increased with the half-life time in rat liver microsomes increased from 34.23 ± 3.33 to 44.15 ± 4.12 min, and the intrinsic rate decreased from 40.49 ± 3.26 to 31.39 ± 2.78 μL/min/mg protein.

**Discussion and conclusions:**

These results indicated that drug–drug interaction might appear during the co-administration of curcumin and amlodipine. The potential mechanism may be due to the inhibition of CYP3A4 by curcumin. Thus, this interaction should be given special attention in the clinic and needs further experiments to characterize the effect in humans.

## Introduction

Curcumin is a kind of polyphenol isolated from *Curcumae Longae Rhizoma Purveratum*, which is usually used as a food colour, but is also used in the clinic for the treatment and prevention of various diseases (Goel et al. 2008). Previously, curcumin was reported to possess a number of pharmacological effects, including antitumor, antioxidant, and anti-inflammatory (Chainoglou and Hadjipavlou-Litina 2019). For example, curcumin can inhibit the growth and invasion of human monocytic leukaemia SHI-1 cells by regulating MAPK and MMP signalling (Zhu et al. 2020), it also can inhibit the progression of breast cancer (Mittal et al. 2020; Pereira et al. 2020). In the clinic, curcumin is always used for the therapy of hyperlipidaemia, which makes it easier to combine with other drugs (Zingg et al. 2013; Panahi et al. 2018).

As hyperlipidaemia and hypertension have many common risk factors and have close associations in many aspects, the clinical therapy of hyperlipidaemia and hypertension always combines different drugs to make the therapy more effective (Ruixing et al. 2009). Amlodipine is one of the most common drugs used for the treatment of hypertension, which is usually co-administrated with other hypolipidemic drugs, such as simvastatin (Fuhrmann et al. 2019). Co-administration of different drugs could induce drug–drug interactions, which can affect the pharmacokinetics of drugs. For example, epigallocatechin-3-gallate can inhibit the metabolism of amlodipine due to the inhibitory effect on the activity of CYP3A4 (Han et al. 2019). Curcumin was demonstrated to be an inhibitor of CYP3A4, which is responsible for the metabolism of amlodipine (Lee et al. 2011; Zhu et al. 2014). Moreover, curcumin can be combined with amlodipine for the treatment of hyperlipidaemia and hypertension. Therefore, it is necessary to investigate the drug–drug interaction between curcumin and amlodipine, which can directly affect the pharmacokinetic and pharmacological effect of amlodipine.

In this study, the drug–drug interaction between curcumin and amlodipine was studied in rats, to explore the effect of curcumin on the pharmacokinetics of amlodipine and its potential mechanism, which can provide more guidance for the clinical co-administration of curcumin and amlodipine.

## Materials and methods

### Materials and reagents

Standards of amlodipine (purity >98%), felodipine (purity >98%) and curcumin (purity >98%) were purchased from the National Institute for the Control of Pharmaceutical and Biological Products (Beijing, China). β-NADPH was obtained from Sigma Chemical Co. (St. Louis, MO). Pooled rat liver microsomes (RLM) were purchased from BD Biosciences Discovery Labware (Woburn, MA). Acetonitrile and methanol were purchased from Fisher Scientific (Fair Lawn, NJ). Formic acid was purchased from Anaqua Chemicals Supply Inc. Limited (Houston, TX). Ultrapure water was prepared with a Milli-Q water purification system (Millipore, Billerica, MA). All other chemicals were of analytical grade or better.

### Animals

Male Sprague–Dawley rats weighing 230–250 g were supplied by Sino-British Sippr/BK Lab Animal Ltd (Shanghai, China). Animals were sustained under 22 ± 2 °C and 50 ± 10% relative humidity in an air-conditioned animal quarter, and acclimatized to the facilities for 5 days. Water and food (laboratory rodent chow, Shanghai, China) were allowed *ad libitum*. Before each experiment, animals were fasted with free access to water for 12 h. The experiment was approved by the Animal Care and Use Committee of Yidu Central Hospital of Weifang.

### LC-MS/MS and conditions

The concentration of amlodipine was analyzed using an Agilent 1290 series liquid chromatography system (Agilent Technologies, Palo Alto, CA) as previously reported (Zhang et al. 2018). Briefly, the sample was separated on Waters Xbridge C18 column (100 × 3.0 mm, i.d.; 3.0 μm, Waters Corporation, Milford, MA) and eluted with an isocratic mobile phase: solvent A (water containing 0.1% formic acid) – solvent B (acetonitrile) (65:35, v/v). The column temperature was set at 25 °C, the flowing rate at 0.4 mL/min and the injection volume at 5 μL. Mass spectrometric detection was carried out on an Agilent 6460 triple-quadruple mass spectrometer (Agilent Technologies, Santa Clara, CA) with Turbo Ion spray, which is connected to the liquid chromatography system. The mass scan mode was MRM positive. The precursor ion and product ion were *m*/*z* 409.2→206.1 for amlodipine, and *m*/*z* 384.5→338.1 for felodipine as the internal standard. The collision energy for amlodipine and felodipine was 30 and 20 eV, respectively. The MS/MS conditions were optimized as follows: fragmentor, 110 V; capillary voltage, 3.5 kV; nozzle voltage, 500 V; nebulizer gas pressure (N2), 40 psig; drying gas flow (N_2_), 10 L/min; gas temperature, 350 °C; sheath gas temperature, 400 °C; sheath gas flow, 11 L/min. Agilent MassHunter B.07 software was used for the control of the equipment and data acquisition. Agilent Quantitative analysis software (Agilent Technologies, Santa Clara, CA) was used for data analysis.

### Pharmacokinetic experiment

The pharmacokinetics of amlodipine was investigated in rats. Twelve rats were divided into two groups randomly, including group A: pre-treated with curcumin and group B: without pre-treatment of curcumin. The amlodipine and curcumin fraction powders were all homogenized in 1.5% Tween 80 aqueous solution with a mortar and pestle. For group A, curcumin was administrated to rats at a dose of 2 mg/kg for 10 days. The dose of amlodipine administrated to both group A and group B was 1 mg/kg. After the administration of amlodipine 0, 0.083, 0.25, 0.5, 1, 2, 3, 4, 6, 8, 12, 24, 36, and 48 h, the plasma samples were collected into a heparinized tube via the oculi chorioideae vein. Plasma samples were centrifuge at 3500 rpm for 10 min, and the supernatant was stored at −80 °C until analysis.

### Preparation of rat plasma samples

To 100 μL aliquot of a plasma sample, 20 μL methanol and 180 μL internal standard methanol solution (2 ng/mL) were added and vortexed for 60 s to mix in a 1.5 mL polypropylene tube, and were centrifuged at 12,000 rpm for 10 min. The supernatant was removed into an injection vial, and a 3 μL aliquot was injected into the LC-MS/MS system for analysis.

### Metabolic experiment with rat liver microsomes

Using rat liver microsomes, the metabolic stability of amlodipine was investigated. The reaction mixture was incubated at 37 °C for 5 min and then amlodipine (1 μM) was added. For the curcumin, curcumin was added into rat liver microsomes before the addition of amlodipine, and preincubating for 30 min at 37 °C. After incubating for 0, 1, 3, 5, 15, 30, and 60 min, aliquots of 30 μL were collected from reaction volumes. Ice-cold acetonitrile (60 μL) containing felodipine was used to terminate the reaction. The sample preparation method was the same as the plasma sample preparation method. The concentration of amlodipine was detected by LC-MS/MS.

The metabolic stability of amlodipine was evaluated by the value of half-life (*t*_1/2_) *in vitro*, calculated by the following equations:

$$t_{1/2}=0.693/k;$$

V (μL/mg) = volume of incubation (μL)/protein in the incubation (mg);

$$\text{Intrinsic}\;\text{clearance}\;\left(\text{Clint}\right)\;\left(\mu L/\text{min}/\text{mg}\;\text{protein}\right)=V\;\times \;0.693/t_{1/2}.$$

### Statistical analysis

All data were presented as mean value ± SD of triple experiments. The pharmacokinetic parameters were calculated using DAS 3.0 pharmacokinetic software (Chinese Pharmacological Association, Anhui, China). Differences between groups were analyzed by one-way ANOVA, and it was considered to be statistical when *p* < 0.05.

## Results

### Curcumin increased the system exposure of amlodipine

**Figure 1.**
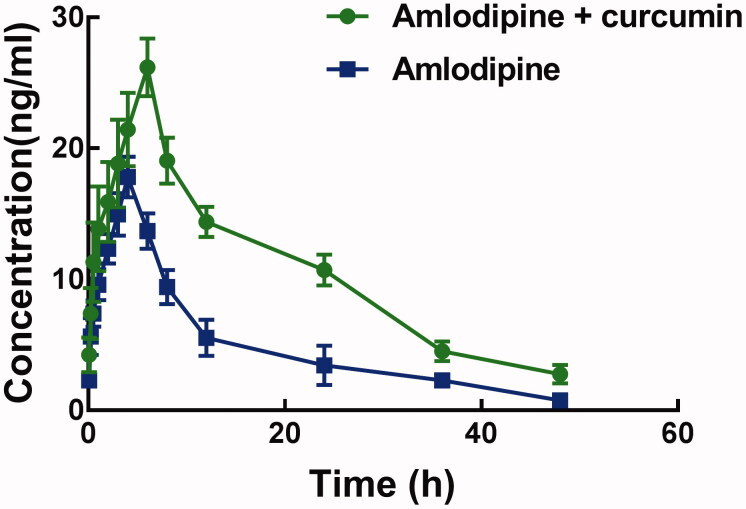
The pharmacolinetic profile of amlodipine with pre-treatment of curcumin (group A) and amlodipine alone (group B) as control.

Figure 1 shows the pharmacokinetic profile of amlodipine with or without pre-treated with curcumin. The administration of curcumin significantly affected the pharmacokinetic profile, as the peak plasma concentration (*C*_max_) and the time reach *C*_max_ was significantly different.

Corresponding pharmacokinetic parameters are summarized in Table 1. The *C*_max_ of amlodipine is 17.80 ± 1.56 μg/L, which was significantly increased to 26.19 ± 2.21 μg/L after pre-treatment with curcumin (*p* < 0.05). Meanwhile, curcumin also increased the *AUC*_(0–t)_ of amlodipine from 238.68 ± 45.59 to 507.27 ± 60.23 μg/h/L significantly (*p* < 0.05).The half-life time (*t*_1/2_) and the *T*_max_ of amlodipine were prolonged by the administration of curcumin, which is consistent with the decrease of clearance rate (1.79 ± 0.26 versus 4.09 ± 0.81 L/h/kg). These changes in the pharmacokinetic parameters of amlodipine indicated an increase in the system exposure of amlodipine.

**Table 1.** Pharmacokinetic parameters of 1 mg/kg amlodipine with pre-treatment of 2 mg/kg curcumin (group A) and amlodipine without any treatment (group B) as control.

	Group A	Group B
AUC_(0–t)_ (μg·h/L)	507.27 ± 60.23	238.68 ± 45.59
*t*_1/2_ (h)	14.96 ± 1.64	11.43 ± 1.20
*T*_max_ (h)	5.87 ± 0.13	3.74 ± 0.16
CL (L/h/kg)	1.79 ± 0.26	4.09 ± 0.81
*C*_max_ (μg/L)	26.19 ± 2.21	17.80 ± 1.56

### Curcumin increased the metabolic stability of amlodipine in rat liver microsomes

To explore the potential mechanism and the metabolic stability of amlodipine, the metabolism of amlodipine was investigated in rat liver microsomes. The half-life of amlodipine in rat liver microsomes was 34.23 ± 3.33 min, while it was significantly prolonged to 44.15 ± 4.12 min after the administration of curcumin (*p* < 0.05). The intrinsic clearance rate was also affected by curcumin. The intrinsic clearance rate of amlodipine was 40.49 ± 3.26 μL/min/mg protein, which significantly decreased to 31.39 ± 2.78 μL/min/mg protein after the administration of curcumin (*p* < 0.05). These results indicated an increase in the metabolic stability of amlodipine, which is consistent with the pharmacokinetic study.

## Discussion

With the development of medical science, co-administration of different kinds of drugs is widely accepted, especially for the treatment of similar diseases. Hyperlipidaemia and have many common risk factors and have close associations in many aspects, these two diseases are often treated together with combination of applicative drugs. Curcumin and amlodipine are common drugs for the treatment of hyperlipidaemia and hypertension, respectively (Maithilikarpagaselvi et al. 2016; Angeli et al. 2018; Panahi et al. 2018), which makes them possible to co-administrate in the clinic. Drug–drug interaction is the main factor that affects the metabolism of drugs, which is closely associated with the pharmaceutical effect or toxicity of drugs. For example, co-administration of triptolide and fenofibrate induces drug-drug interaction, which inhibits the metabolism of fenofibrate (Li et al. 2019).

In this study, we found the pharmacokinetic profile of amlodipine was affected, as the AUC_(0–t)_, *t*_1/2_, and other pharmacokinetic parameters were changed after combining with curcumin. These results indicated that curcumin inhibited the metabolism of amlodipine. Furthermore, the intrinsic clearance rate of amlodipine in rat liver microsomes was decreased by curcumin, which suggested an increase in the metabolic stability of amlodipine. Previously, inhibition or induction of cytochrome P450 enzymes (CYP450) was reported to be the main cause responsible for the drug-drug interaction. Especially, the activity of CYP3A4, an enzyme in the liver involved in the metabolism of various drugs, plays vital roles during the pharmacokinetics of several drugs, such as puerarin, warfarin, and asiatic acid, where the inhibition on CYP3A4 resulted in the drug–drug interaction (Guo et al. 2018; Liu et al. 2019; Song et al. 2020). It has been reported that amlodipine is the substrate of CYP3A4, and curcumin has been demonstrated to be an inhibitor of CYP3A4 (Lee et al. 2011; Zhu et al. 2014). Therefore, we speculated the effect of curcumin on the pharmacokinetics of amlodipine was a result of the inhibition of CYP3A4. Additionally, similar drug–drug interaction may occur between amlodipine and drugs that affect the activity of CYP3A4 or curcumin and drugs metabolized by CYP3A4. These potential interactions need more *in vivo* or *in vitro* experiments to identify.

However, this is an *in vivo* study in rat provided evidence for the potential drug-drug interaction between curcumin and amlodipine, which cannot represent the interaction in human. Therefore, more experiments that more closely resemble human metabolism are needed in future studies.

## Conclusions

Co-administration of curcumin and amlodipine may induce drug–drug interaction, which increases the system exposure of amlodipine due to the inhibition of CYP3A4. Therefore, the combination of these two drugs should be given special attention in the clinic. There are a number of drugs that affect the activity of CYP3A4 or other CYPs. Potential drug–drug interactions might appear during co-administration, which needs further studies to verify.

## References

[CIT0001] Angeli F, Trapasso M, Signorotti S, Verdecchia P, Reboldi G. 2018. Amlodipine and celecoxib for treatment of hypertension and osteoarthritis pain. Expert Rev Clin Pharmacol. 11(11):1073–1084. 30362840 10.1080/17512433.2018.1540299

[CIT0002] Chainoglou E, Hadjipavlou-Litina D. 2019. Curcumin analogues and derivatives with anti-proliferative and anti-inflammatory activity: structural characteristics and molecular targets. Expert Opin Drug Discov. 14(8):821–842. 31094233 10.1080/17460441.2019.1614560

[CIT0003] Fuhrmann S, Koppen A, Seeling A, Knoth H, Schroder J. 2019. Analysis of secondary care data to evaluate the clinical relevance of the drug–drug interaction between amlodipine and simvastatin. Z Evid Fortbild Qual Gesundhwes. 146:21–27. 31324418 10.1016/j.zefq.2019.06.003

[CIT0004] Goel A, Kunnumakkara AB, Aggarwal BB. 2008. Curcumin as “Curecumin”: from kitchen to clinic. Biochem Pharmacol. 75(4):787–809. 17900536 10.1016/j.bcp.2007.08.016

[CIT0005] Guo L, Cui Y, Hao K. 2018. Effects of glycyrrhizin on the pharmacokinetics of asiatic acid in rats and its potential mechanism. Pharm Biol. 56(1):119–123. 29357733 10.1080/13880209.2018.1428634 PMC6130451

[CIT0006] Han X, Zhang H, Hao H, Li H, Guo X, Zhang D. 2019. Effect of epigallocatechin-3-gallate on the pharmacokinetics of amlodipine in rats. Xenobiotica. 49(8):970–974. 30182817 10.1080/00498254.2018.1519732

[CIT0007] Lee CK, Ki SH, Choi JS. 2011. Effects of oral curcumin on the pharmacokinetics of intravenous and oral etoposide in rats: possible role of intestinal CYP3A and P-gp inhibition by curcumin. Biopharm Drug Dispos. 32(4):245–251. 21506134 10.1002/bdd.754

[CIT0008] Li T, Liu J, Zheng Y, Yang S, Liu X, Li X. 2019. Effects of triptolide on pharmacokinetics of fenofibrate in rats and its potential mechanism. Xenobiotica. 49(2):211–215. 29412757 10.1080/00498254.2018.1438685

[CIT0009] Liu L, Li P, Qiao L, Li X. 2019. Effects of astragaloside IV on the pharmacokinetics of puerarin in rats. Xenobiotica. 49(10):1173–1177. 29790819 10.1080/00498254.2018.1480819

[CIT0010] Maithilikarpagaselvi N, Sridhar MG, Swaminathan RP, Sripradha R, Badhe B. 2016. Curcumin inhibits hyperlipidemia and hepatic fat accumulation in high-fructose-fed male Wistar rats. Pharm Biol. 54(12):2857–2863. 27241764 10.1080/13880209.2016.1187179

[CIT0011] Mittal L, Aryal UK, Camarillo IG, Raman V, Sundararajan R. 2020. Effective electrochemotherapy with curcumin in MDA-MB-231-human, triple negative breast cancer cells: a global proteomics study. Bioelectrochemistry. 131:107350. 31518962 10.1016/j.bioelechem.2019.107350

[CIT0012] Panahi Y, Ahmadi Y, Teymouri M, Johnston TP, Sahebkar A. 2018. Curcumin as a potential candidate for treating hyperlipidemia: a review of cellular and metabolic mechanisms. J Cell Physiol. 233(1):141–152. 28012169 10.1002/jcp.25756

[CIT0013] Pereira MC, Mohammed R, VAN Otterlo WAL, DE Koning CB, Davids H. 2020. *In vitro* analysis of the combinatory effects of novel aminonaphthoquinone derivatives and curcumin on breast cancer progression. Anticancer Res. 40(1):229–238. 31892571 10.21873/anticanres.13944

[CIT0014] Ruixing Y, Jinzhen W, Weixiong L, Yuming C, Dezhai Y, Shangling P. 2009. The environmental and genetic evidence for the association of hyperlipidemia and hypertension. J Hypertens. 27(2):251–258. 19155782 10.1097/HJH.0b013e32831bc74d

[CIT0015] Song J, Dai H, Zhang H, Liu Y, Zhang W. 2020. Influence of glycyrrhetinic acid on the pharmacokinetics of warfarin in rats. Xenobiotica. 50(5):602–605. 31542982 10.1080/00498254.2019.1671637

[CIT0016] Zhang C, Gao Z, Niu L, Chen X. 2018. Effects of triptolide on pharmacokinetics of amlodipine in rats by using LC-MS/MS. Pharm Biol. 56(1):132–137. 29385884 10.1080/13880209.2018.1430835 PMC6130517

[CIT0017] Zhu G, Shen Q, Jiang H, Ji O, Zhu L, Zhang L. 2020. Curcumin inhibited the growth and invasion of human monocytic leukaemia SHI-1 cells in vivo by altering MAPK and MMP signalling. Pharm Biol. 58(1):25–34. 31854220 10.1080/13880209.2019.1701042 PMC6968541

[CIT0018] Zhu Y, Wang F, Li Q, Zhu M, Du A, Tang W, Chen W. 2014. Amlodipine metabolism in human liver microsomes and roles of CYP3A4/5 in the dihydropyridine dehydrogenation. Drug Metab Dispos. 42(2):245–249. 24301608 10.1124/dmd.113.055400

[CIT0019] Zingg JM, Hasan ST, Meydani M. 2013. Molecular mechanisms of hypolipidemic effects of curcumin. Biofactors. 39(1):101–121. 23339042 10.1002/biof.1072

